# A Comparative Study of Computational Methods for Compressed Sensing Reconstruction of EMG Signal

**DOI:** 10.3390/s19163531

**Published:** 2019-08-13

**Authors:** Lorenzo Manoni, Claudio Turchetti, Laura Falaschetti, Paolo Crippa

**Affiliations:** DII—Dipartimento di Ingegneria dell’Informazione, Università Politecnica delle Marche, Via Brecce Bianche 12, I-60131 Ancona, Italy

**Keywords:** compressed sensing, signal reconstruction, surface electromyography, biosignal, sensors, wireless sensor networks

## Abstract

Wearable devices offer a convenient means to monitor biosignals in real time at relatively low cost, and provide continuous monitoring without causing any discomfort. Among signals that contain critical information about human body status, electromyography (EMG) signal is particular useful in monitoring muscle functionality and activity during sport, fitness, or daily life. In particular surface electromyography (sEMG) has proven to be a suitable technique in several health monitoring applications, thanks to its non-invasiveness and ease to use. However, recording EMG signals from multiple channels yields a large amount of data that increases the power consumption of wireless transmission thus reducing the sensor lifetime. Compressed sensing (CS) is a promising data acquisition solution that takes advantage of the signal sparseness in a particular basis to significantly reduce the number of samples needed to reconstruct the signal. As a large variety of algorithms have been developed in recent years with this technique, it is of paramount importance to assess their performance in order to meet the stringent energy constraints imposed in the design of low-power wireless body area networks (WBANs) for sEMG monitoring. The aim of this paper is to present a comprehensive comparative study of computational methods for CS reconstruction of EMG signals, giving some useful guidelines in the design of efficient low-power WBANs. For this purpose, four of the most common reconstruction algorithms used in practical applications have been deeply analyzed and compared both in terms of accuracy and speed, and the sparseness of the signal has been estimated in three different bases. A wide range of experiments are performed on real-world EMG biosignals coming from two different datasets, giving rise to two different independent case studies.

## 1. Introduction

Surface electromyography (sEMG) is a technique to capture and measure the electrical potential at the skin surface due to muscle activity [1,2]. The registered EMG signal in a muscle is the collective action potential of all muscular fibers of the motor unit that work together since they are stimulated by the same motor neuron. The muscular contraction is generated by a stimulus that propagates from the brain cortex to the target muscle as an electrical potential, named action potential (AP). sEMG signal is frequently used for the evaluation of muscle functionality and activity, thanks to the non-invasiveness and ease of this technique [3,4,5]. Common applications are fatigue analysis [6] of rehabilitation exercises [5,7], postural control [8], musculoskeletal disorder analysis [9], gait analysis [10], movement recognition [11], gesture recognition [12], prosthetic control [13,14,15], to cite only a few. Among these applications monitoring and automatic recognition of human activities are of particular interests both for sport and fitness as well as for healthcare of elderly and impaired people [16,17]. Wireless body area networks (WBANs) provide an effective and a relatively low-cost solution for biosignal monitoring in real time [18,19]. A WBAN typically consists of one or more low-power, miniaturized, lightweight devices with wireless communication capabilities that operate in the proximity of a human body [20]. However, power consumption represents a major problem for the design and for the widespread of such devices. A large part of the device power consumption is required for the wireless transmission of the signals which are recorded from multiple channels at a high-sampling rate [21]. Standard compression protocols have a high computational complexity and the implementation in the sensor nodes would add a big overhead to the power consumption. Compressed sensing (CS) techniques that lie on the sparsity property of many natural signals, have been successfully applied in the WBAN long term signal monitoring, since CS significantly saves the transmit power by reducing the sampling rate [22,23,24,25,26]. Recent studies have applied CS to sEMG signals for gesture recognition, an innovative application field of the sEMG signal analysis [27,28]. In this context, CS has a great importance in reducing the size of transmitted sEMG data while being able to reconstruct good quality signals and recognize hand movements. The fundamental idea behind CS is that rather than first sampling at a high rate and then compressing the sampled data, as usually done in standard techniques, we would like to find ways to directly sense the data in compressed form, i.e., at a lower sampling rate. To make this possible, CS relies on the concept of sparsity which implies that certain classes of signals, sparse signals, when expressed in a proper basis have only a small number of non-zero coordinates. The CS field grew out of the seminal work of Candes, Romberg, Tao and Donoho [29,30,31,32,33,34,35], who showed that a finite dimensional sparse signal can be recovered from several samples much smaller that its length. The CS paradigm combines two fundamental stages, encoding and reconstruction. The reconstruction of a signal acquired with CS represents the most critical and expensive stage as it involves an optimization which seeks the best solution to an undetermined set of linear equations with no prior knowledge of the signal except that it is sparse when represented in a proper basis. To obtain the better performance in the reconstruction of the undersampled signal a large variety of algorithms have been developed in recent years [36]. In the class of computational techniques for solving sparse approximation problems, two approaches are computationally practical and lead to provably exact solutions under some defined conditions: convex optimization and greedy algorithms. Convex optimization is the original CS reconstruction algorithm formulated as a linear programming problem [37]. Unlike convex optimization, greedy algorithms try to solve the reconstruction problem in a less exact manner. In this class, the most common algorithms used in practical applications are orthogonal matching pursuit (OMP) [38,39,40,41,42], compressed sampling matching pursuit (CoSaMP) [43,44], normalized iterative hard thresholding (NIHT) [45]. All these algorithms are applicable in principle to a generic signal; however, in the design and implementation of a sensor architecture is of paramount importance to assess the performance with reference to the specific signal to be acquired. Additionally, the performance of the algorithms can vary very widely, so that a comparative study that demonstrates the practicability of such algorithms are welcomed by designers of low powers WBANs for biosignal monitoring [26].

The aim of this paper is to explore the trade-off in the choice of a compressed sensing algorithm, belonging to the classes of techniques previously described, to be applied in EMG sensor-applications. Thus, the ultimate goal of the paper is to present a comparative study of computational methods for CS reconstruction of EMG signals, in real-world EMG signal acquisition systems, leading to efficient, low-power WBANs. For example, a useful application of this comparative study can be the selection of the best algorithm to be applied in EMG-based gesture recognition. In addition, the effect of this basis used for reconstruction on signal sparseness has been analyzed for three different bases.

This paper is organized as follows. Section 2 summarizes the basic concept of CS theory. Section 3 is mainly focused on CS reconstruction algorithms and in particular gives a complete description of four algorithms: Convex Optimization, OMP, CoSaMP, and NIHT. Section 4 reports a comparative study of the four algorithms performance when applied to real-world EMG signals.

## 2. Compressed Sensing Background

In this section, we provide an overview of the basic concepts of the CS theory. In Table 1, for ease of reference, a list of the main symbols and definitions used throughout the text are reported. Some of these are currently adopted in the literature while other specific operators will be defined later.

CS theory asserts that rather than acquire the entire signal and then compress, it is usually done in compression techniques, it is possible to capture only the useful information at rates smaller than the Nyquist sampling rate.

The CS paradigm combines two fundamental stages, encoding and reconstruction.

In the encoding stage the *N*-dimensional input signal *f* is encoded into a *M*-dimensional set of measurements *y* through a linear transformation by the M×N measurement matrix Φ where y=Φf. In this way with M<N the CS data acquisition system directly translates analog data into a compressed digital form.

In the reconstruction stage given by f=Ψx, assuming the signal *f* to be recovered is known to be sparse in some basis $\Psi =[\Psi_{1},\ldots ,\Psi_{N}]$, in the sense that all but a few coefficients $x_{i}$ are zero, the sparsest solution *x* (fewest significant non-zero $x_{i}$) is found. The reconstruction algorithms exhibit better performance when the signal to be reconstructed is exactly *k*-sparse on the basis Ψ, i.e., with $x_{i}\neq 0$ for i∈Λ,|Λ|=k. Thus, in some algorithms the N−k elements of *x* that give negligible contributions are discarded. To this end the following operator is defined

(1) $$\Lambda =\underset{k,\Psi }{supp}\left(x\right):\;|\Lambda |=k,\;\gamma_{i}>\gamma_{j},\gamma_{i}=\left|x_{i}\right|{∥\Psi_{i}∥}_{2},\;\;\mathrm{for}\;i\in \Lambda ,\;j\notin \Lambda$$

that selects the set Λ of *k* indexes corresponding to largest values $|x_{i}|{∥\Psi_{i}∥}_{2}$. The set Λ so derived represents the so-called *set of sparsity*. Another useful definition in this context is the operator F(x,Λ) that returns a vector with the same elements of *x* in the sub-set Λ and zero elsewhere, formally

(2) $$F{\left(x,\Lambda \right)}_{\Lambda }=x_{\Lambda },\;F{\left(x,\Lambda \right)}_{I_{N}\setminus \Lambda }=0,\;I_{N}=\left\{1,2,\ldots ,N\right\}\;,$$

where $I_{N}\setminus \Lambda$ means difference of the two sets $I_{N}$ and Λ. The consecutive application of the two operators gives rise to a *k*-sparse vector obtained from *x* by keeping only the components with the largest values of $|x_{i}|{∥\Psi_{i}∥}_{2}$, and will be synthetically denoted by ${\left[x\right]}_{k}$, called *reduced operator*. Thus

(3) $${\left[x\right]}_{k}=F\left(x,\underset{k,\Psi }{supp}\left(x\right)\right).$$

A natural formulation of the recovery problem is within an $l_{0}$-norm minimization framework, which seeks a solution *x* of the problem

(4) $$\min_{x\in R^{N}}{∥x∥}_{0}\;\;subjectto\;\;y=\Phi \Psi x\;,$$

where ${∥\cdot ∥}_{0}$ is a counting function that returns the number of non-zero components in its argument. Unfortunately, the $l_{0}$-minimization problem is NP-hard, and hence cannot be used for practical application. A method to avoid using this computationally intractable formulation is to consider an $l_{1}$-minimization problem.

It has been shown [33] that when *x* is the solution to the convex approximation problem

(5) $$\min_{x\in R^{N}}{∥x∥}_{1}\;\;subjectto\;\;y=\Phi \Psi x$$

then the reconstruction f=Ψx is exact. More specifically only *M* measurements in the Φ domain selected uniformly at random, are needed to reconstruct the signal provided *M* satisfies the inequality

(6) $$M\geq C\nu^{2}\left(\Phi ,\Psi \right)S\log N$$

where *N* represents the signal size, *S* the index of sparseness, *C* a constant and ν(Φ,Ψ) the coherence between the sensing basis Φ and the representation basis Ψ. Coherence measures the largest correlation between any two elements of Φ and Ψ and is given by $\nu \left(\Phi ,\Psi \right)=\max_{k,j}\left|\langle \Phi_{k},\Psi_{j}\rangle \right|$, with $\nu^{2}\left(\Phi ,\Psi \right)\in \left[0,N\right]$. Random matrices are largely incoherent (ν=1) with any fixed basis Ψ. Therefore, as the smaller the coherence the fewer samples are needed, random matrices are the best choice for sensing basis.

The usually adopted performance metric to measure the reduction in the data required to represent the signal *f* is the compression ratio CR defined as

(7) $$CR=\frac{N}{M}\;,$$

that is the ratio between the length of the original and compressed signal vectors. Instead sparsity is usually defined as

(8) $$S_{N}=\frac{k}{N}.$$

Sometimes is more convenient to define sparsity with respect to dimension *M*, thus giving $S_{M}=k/M$. Obviously the two equation are related by $S_{M}=\mathrm{CR}\;S_{N}$.

## 3. The Algorithms

As the CS sampling framework includes two main activities, encoding and reconstruction, some specific algorithms must be derived for this purpose.

### 3.1. Encoding

CS encoder uses a linear transformation to project the vector *f* into the lower dimensional vector *y*, through the measurement matrix Φ. In addition of being incoherent with respect to the basis Ψ, measurement matrix Φ must facilitate encoding practical implementation. One widely used approach is to use Bernoulli random matrices Φ(i,j)=±1. This choice allows saving of multiplication in the matrix-product operation y=Φf. Moreover, simple, fast and low-power digital and analog hardware implementations of the encoder are possible [26].

### 3.2. Basis Matrix Ψ

A wide range of basis matrices Ψ can be adopted in Equation (4), three of the most familiar bases will be used in this paper, namely DCT, Haar and Daubechies’ wavelet (DBW). Although DCT seems not to be an adequately sparse basis for EMG signal it was used in one of the two case studies because of signal pre-filtering during acquisition as it will be explained in Section 4. Additionally, other recent works [46,47] have demonstrated the validity of DCT basis for CS applied to EMG signal. Matrix Ψ for Haar and DB4 was built using parallel filter bank wavelet implementation [48].

### 3.3. Reconstruction

CS reconstruction algorithms can be divided into two classes: convex optimization and greedy algorithms.

#### 3.3.1. Convex Optimization

##### L1-minimization

The CS theory asserts that when *f* is sufficiently sparse, the recovery via $l_{1}$-minimization is provably exact. Thus, a fundamental algorithm for reconstruction is the convex optimization wherein the $l_{1}$-norm of the reconstructed signal is an efficient measure of sparsity. The CS reconstruction process is described by Equation (5) which can be regarded as a linear programming problem. This approach is also known as basis pursuit.

By assuming $f\left(k\right)=\left[f(kN-N+1),\ldots ,f(kN)\right],\;k=1,\ldots ,L$ is a frame of the EMG signal to be reconstructed, $\Psi =\left[\Psi_{1},\ldots ,\Psi_{N}\right]$ is an N×N basis matrix, Φ an M×N Bernoulli matrix, then the constraint in Equation (5) can be rewritten as

(9) y=ΦΨx=Ax

with A=ΦΨ. Introducing the Lagrange function

(10) $$L\left(x,\lambda \right)={∥x∥}_{1}+\lambda^{T}\left(Ax-y\right)$$

where *T* denotes matrix transposition thus to solve the problem (5) is equivalent to determine the stationary point of L(x,λ) with respect to both *x* and λ. A usual technique for this problem is the projected-gradient algorithm based on the following iterative scheme

(11) $$x_{t+1}=x_{t}-\mu \frac{\partial L}{\partial x}{|}_{x_{t}}$$

where μ is a parameter that regulates the convergence of the algorithm. By deriving (10) we obtain

(12) $$\frac{\partial L}{\partial x}=sgn\left(x\right)+A^{T}\lambda \;,$$

then combining Equations (11) and (12) with constraint $Ax_{t}=y$ and assuming ${\left(AA^{T}\right)}^{-1}$ is invertible, it results

(13) $$\lambda =-{\left(AA^{T}\right)}^{-1}A\;sgn\left(x_{t}\right)\;.$$

Finally, the following iterative solution

(14) $$x_{t+1}=x_{t}-\mu \left(I-A^{T}{\left(AA^{T}\right)}^{-1}A\right)\;sgn\left(x_{t}\right)$$

is obtained. To make the convergence parameter μ independent of signal power the following normalized version of the algorithm can be adopted

(15) $$x_{t+1}=x_{t}-\mu \frac{{∥x_{t}∥}_{1}}{N}P\;sgn\left(x_{t}\right)$$

with $P=I-A^{†}A$ and $A^{†}=A^{T}{\left(AA^{T}\right)}^{-1}$. To initialize the algorithm a vector $x_{0}$ given by

(16) $$x_{0}=A^{†}y$$

that solves the following $l_{2}$-minimization problem

(17) $$x_{0}=\arg\min_{x}{∥x∥}_{2}^{2}\;\;s.t.\;\;Ax=y$$

has been chosen. The parameter μ determines the convergence of the algorithm; thus, to establish a proper choice of its value a convergence criterion should be derived. However, a complete treatment of convergence is a difficult task and is out of the scope of this paper. To face this problem the value of μ has been chosen using a semi-heuristic criterion that bounds the steady-state ripple given by

(18) $$\frac{{∥x_{t+1}-x_{t}∥}_{2}}{{∥x_{t}∥}_{2}}<\epsilon_{max},\;t\to \infty$$

where $\epsilon_{max}$ specifies the desired accuracy. In such a way we obtain

(19) $$\mu \leq \frac{\epsilon_{max}N}{∥P\;sgn{(x_{\infty }∥}_{2}}\;\frac{{∥x_{\infty }∥}_{2}}{{∥x_{0}∥}_{1}}<\frac{\epsilon_{max}N}{{∥P\;sgn\left(x_{\infty }\right)∥}_{2}}$$

which can be reduced to the more practical condition

(20) $$\mu \leq \frac{\epsilon_{max}N}{{∥P\;sgn\left(x_{0}\right)∥}_{2}}\;.$$

An optimized version of the algorithm with a reduced number of products can be derived as follows. Let us rewrite Equation (15) as

(21) $$x_{t+1}=x_{t}-\mu \frac{{∥x_{t}∥}_{1}}{N}q_{t}$$

where

(22) $$q_{t}=P_{s_{t}}=\sum_{j=1}^{N}P_{j}s_{t}\left(j\right)$$

and

(23) $$s_{t}=\left(s_{t}\left(1\right),\ldots ,s_{t}\left(N\right)\right)=sgn\left(x_{t}\right).$$

The variation of *q* from (t−1) to *t*

(24) $$q_{t}-q_{t-1}=\sum_{j=1}^{N}P_{j}\left[s_{t}\left(j\right)-s_{t-1}\left(j\right)\right]$$

only depends on

(25) $$\Delta s_{t}\left(j\right)=s_{t}\left(j\right)-s_{t-1}\left(j\right)=\left\{\begin{array}{cc} 2 & \mathrm{for}\;\;s_{t-1}\left(j\right)=-1,\;s_{t}\left(j\right)=1\\ -2 & \mathrm{for}\;\;s_{t-1}\left(j\right)=1,\;s_{t}\left(j\right)=-1\\ 0 & \mathrm{for}\;\;s_{t-1}\left(j\right)=s_{t}\left(j\right) \end{array}\right.\;\;j=1,\ldots ,N,$$

which can be rewritten in a compact form as

(26) $$\Delta s_{t}\left(j\right)=2s_{t}\left(j\right)v_{t}\left(j\right),\;j=1,\ldots ,N$$

where

(27) $$v_{t}\left(j\right)=\left\{\begin{array}{cc} 1 & \mathrm{for}\;\;s_{t}\left(j\right)\neq s_{t-1}\left(j\right)\\ 0 & \mathrm{for}\;\;s_{t}\left(j\right)=s_{t-1}\left(j\right) \end{array}\right..$$

By defining

(28) $$w_{t}\left(j\right)=\left(s_{t}\left(j\right)+1\right)/2\in \left\{0,1\right\}\;,$$

Equation (27) is equivalent to

(29) $$v_{t}\left(j\right)=w_{t}\left(j\right)⊕w_{t-1}\left(j\right)\;.$$

Finally, from Equations (24), (26) and (29) and defining the set $\Omega_{t}=\left\{j/v_{t}\left(j\right)=1\right\}$ we have

(30) $$q_{t}=q_{t-1}+2\sum_{j\in \Omega_{t}}P_{j}s_{t}\left(j\right)\;.$$

Thus, the summation in Equation (30) is extended only to the terms for which a sign changing from $s_{t-1}$ to $s_{t}$ occurs, thus reducing the number of products required at each step.

A pseudo-code of the L1 algorithm is reported as Algorithm 1.
**Algorithm 1** L1-minimization Input: A=ΦΨ,y,k Inizialize: $\left\{\begin{array}{c} P=\left[p_{1}\ldots p_{N}\right],\;P=I-A^{†}A,\\ x_{0}=A^{†}y\\ t=0 \end{array}\right.$ Output: *k*-sparse coefficient vector *x* **while**
$t<N_{iter}$
**do**  $s_{t}=sgn\left(x_{t}\right),\;w_{t}=\left(s_{t}+1\right)/2$
* // reduce $s_{t}$ to binary vector $w_{t}$*
  **if**
t>0
**then**   $v_{t}=w_{t}⊕w_{t-1}$   $\Omega_{t}=\left\{j/v_{t}\left(j\right)=1\right\}$
* // define the set of indices $\Omega_{t}$ corresponding to a change from $s_{t-1}$ to $s_{t}$*
   $q_{t}=q_{t-1}+2\sum_{j\in \Omega_{t}}p_{j}s_{t}\left(j\right)$  **else**   $q_{t}=Ps_{0}$   $\mu =\frac{\epsilon_{max}N}{{∥q_{0}∥}_{2}}$  **end if**  $x_{t+1}=x_{t}-\mu \frac{{∥x_{t}∥}_{1}}{N}q_{t}$  t=t+1 **end while**

#### 3.3.2. Greedy Algorithms

##### Orthogonal Matching Pursuit (OMP)

This algorithm solves the reconstruction of k−sparse coefficient vector *x*, i.e., with $x_{i}\neq 0,i\;\in \;\Lambda ,\;\left|\Lambda \right|=k$. The algorithm tries to find the *k* directions corresponding to the non-zero *x*-components, starting from the residual $r_{0}$ given by the measurements *y*. At each step t≤k the column $a_{j}$ of *A* that is mostly correlated with the residual is derived. Then the best coefficients $x_{t}$ are found by solving the following $l_{2}$-minimization problem

(31) $$x_{t}=\underset{x}{\arg\min}{∥y-A_{t}x∥}_{2},$$

thus giving

(32) $$x_{t}=A_{t}^{†}y.$$

Finally, the residual, the difference between the actual measure and the A mathematical description of the algorithm is reported in Algorithm 2.
**Algorithm 2** OMP Input: A=ΦΨ,y,k Initialize: $\left\{\begin{array}{cc} r_{0}=y & //residual\\ A_{0}=\emptyset & //columns\\ t=0 & \end{array}\right.$ Output: *k*-sparse coefficient vector *x* ** while**t<k
**do**
  $\lambda_{t}=\underset{j}{\arg\max}\left|a_{j}^{T}r_{t-1}\right|$
* // find the column of A that is most strongly correlated with the residual*  $A_{t}=\left[A_{t-1}\;a_{\lambda_{t}}\right]$
* // merge the new column*  $x_{t}=A_{t}^{†}y\;$
* // find the best coefficients $x_{t}$ from (31)*  $r_{t}=y-A_{t}x_{t}$
* // update the residual*  t=t+1 **end while**

##### Compressive Sampling Matching Pursuit (CoSaMP)

Differently from OMP, the CoSaMP algorithm tries to find the $k_{S}$ columns of $A^{T}$ that are the most strongly correlated with the residual, thus making a correction of the reconstruction on the basis of residual achieved at each step. The $k_{S}$ columns are determined by the selection step

(33) $$W_{t}=\underset{\left|W\right|=k_{S}}{\arg\max}\;{∥A_{W}^{T}\;r_{t}∥}_{1}$$

where $A_{W}^{T}$ is the sub-matrix of $A^{T}$ made by columns indexed by the set *W*, and $r_{t}$ is the residual at current iteration step *t*. Thus, the algorithm proceeds to estimate the best coefficients *h* for approximating the residual with the new columns indexed by $T_{t}=\Lambda_{t}\cup W_{t}$. As this step corresponds to an LMS problem, it results

(34) $$h=A_{T_{t}}^{†}y\;.$$

Finally, the sparsity operator

(35) $$x=F(h,\Lambda_{t+1})$$

with $\Lambda_{t+1}=\underset{k,\Psi }{supp(h)}$ is applied to obtain the sparse vector *x*. At the end of the algorithm the residual is updated with the new signal reconstruction $A_{\Lambda_{t+1}}\;x_{\Lambda_{t+1}}$. A pseudo-code of the algorithm is reported in Algorithm 3.

##### Normalized Iterative Hard Thresholding (NIHT)

The basic idea that underlies NIHT algorithm is that the sparse components to be identified give a large contribution to the gradient of residual. The algorithm tries to find these components by following the gradient of residual $r_{t}=Ax_{t}-y$, i.e.,

(36) $${\tilde{x}}_{t+1}=x_{t}-\mu_{t}\frac{\partial {∥r_{t}∥}_{2}^{2}}{\partial x_{t}}\;,$$

thus obtaining

(37) $${\tilde{x}}_{t+1}=x_{t}-\mu_{t}A^{T}\left(Ax_{t}-y\right)\;.$$

The sparse vector $x_{t+1}$ is derived at each iteration *t* by applying the reduced operator to the estimated vector $x_{t+1}$,

(38) $$\Lambda_{t+1}=\underset{k,\Psi }{supp}\left({\tilde{x}}_{t+1}\right)$$

(39) $$x_{t+1}=F\left({\tilde{x}}_{t+1},\Lambda_{t+1}\right)\;.$$

**Algorithm 3** CoSaMP Input: A=ΦΨ,y,k Initialize: $\left\{\begin{array}{cc} r_{0}=y & //residual\\ t=0 & \end{array}\right.$ $\Lambda_{0}=\underset{|\Lambda |=k}{\arg\max}{∥A_{\Lambda }^{T}\;r_{0}∥}_{1}$
* // find k columns of $A^{T}$ that are most strongly correlated with residual $r_{0}$* Output: *k*-sparse coefficient vector *x*
 **while**
$t<N_{iter}$
**do**
  $k_{S}=\gamma k,\;\gamma \in \left[0,1\right]$
* // number of new columns to be selected*  $W_{t}=\underset{\left|W\right|=k_{S}}{\arg\max}{∥A_{W}^{T}\;r_{t}∥}_{1}$
* // find $k_{S}$ columns of $A^{T}$ that are most strongly correlated with residual $r_{t}$*  $T_{t}=\Lambda_{t}\cup W_{t}$
* // merge the new columns such that $|T_{t}|=k+k_{S}$*  $h=A_{T_{t}}^{†}y$
* // find the best coefficients for residual approximation*
  $\Lambda_{t+1}=\underset{k,\Psi }{supp}\left(h\right)$
* // find the set of sparsity $\Lambda_{t+1}$*
  $x=F(h,\Lambda_{t+1})$
* // find sparse vector x*  $r_{t+1}=y-A_{\Lambda_{t+1}}x_{\Lambda_{t+1}}$
* // update the residual*  t=t+1 **end while**

As in CoSaMP the initialization is made by choosing the columns of $A^{T}$ that are most strongly correlated with residual

(40) $$\Lambda_{0}=\underset{|\Lambda |=k}{\arg\max}\;{∥A_{\Lambda }^{T}\;y∥}_{1}$$

and then estimating the best coefficients

(41) $$x_{0}=A_{\Lambda_{0}}^{†}\;y$$

for residual approximation. A different step size has been used for each $x_{t}$ component by defining the step size vector ρ as

(42) $$\rho =\min_{j}{∥a_{j}∥}_{2}\;\left(\frac{1}{{∥a_{1}∥}_{2}},\;\ldots ,\frac{1}{{∥a_{N}∥}_{2}}\right)\;,$$

thus, normalizing the components of the gradient vector $A^{T}\left(Ax_{t}-y\right)$. In this way the updated equation for *x* becomes

(43) $${\tilde{x}}_{t+1}=x_{t}-\mu_{t}q_{t}$$

where $q_{t}=\rho ⊙A^{T}\left(Ax_{t}-y\right)$ is the normalized gradient vector and ⊙ denotes the element-wise product of vectors. The value of $\mu_{t}$ has been estimated by minimizing the residual, i.e., such that

(44) $$\frac{\partial }{\partial \mu_{t}}{∥Ax_{t+1}-y∥}_{2}^{2}=0$$

or

(45) $$\frac{\partial }{\partial \mu_{t}}{∥A\left(x_{t}-\mu_{t}q_{t}\right)-y∥}_{2}^{2}=0\;.$$

A closed form of $\mu_{t}$ cannot be derived as it depends on the set $\Lambda_{t+1}$ selected after the updating of ${\tilde{x}}_{t+1}$. To circumvent this problem an iterative approach has been used, starting from an initial estimation $\Lambda_{t+1}^{★}$ of $\Lambda_{t+1}$ to compute $\mu_{t}\left(\Lambda_{t+1}^{★}\right)$ and then updating $\Lambda_{t+1}^{★}$ to the true value. In this way from previous Equation (45) we obtain

(46) $$\mu_{t}=\frac{q_{\Lambda_{t+1}^{★}}^{T}\;A^{T}\;\left(Ax_{\Lambda_{t+1}^{★}}-y\right)}{q_{\Lambda_{t+1}^{★}}^{T}\;A^{T}\;Aq_{\Lambda_{t+1}^{★}}}=\frac{w^{T}\epsilon }{w^{T}w}$$

where

(47) $$\begin{array}{c} w=Aq_{\Lambda_{t+1}^{★}}\;\epsilon =Ax_{\Lambda_{t+1}^{★}}-y\\ \Lambda_{t+1}^{★}=\underset{k,\Psi }{supp}\left(x_{t}-\mu_{t}q\right). \end{array}$$

**Algorithm 4** NIHT Input: A=ΦΨ,y,k Initialize: $\left\{\begin{array}{cc} \Lambda_{0}=\underset{|\Lambda |=k}{\arg\max}{∥A_{\Lambda }^{T}\;y∥}_{1} & //find\;the\;columns\;ofA^{T}\;that\;are\;the\\ & //\;most\;strongly\;correlated\;with\;residual\\ x_{0}=A_{\Lambda_{0}}^{†}y & //\;find\;the\;best\;coefficients\;for\;residual\;approximation\\ t=0 & \end{array}\right.$ Output: *k*-sparse coefficient vector *x* **while**$t<N_{iter}$**do**  $r_{t}=Ax_{t}-y$* // update residual*  $\rho =\min_{j}{∥a_{j}∥}_{2}\;\left(\frac{1}{{∥a_{1}∥}_{2}},\;\ldots ,\frac{1}{{∥a_{N}∥}_{2}}\right)$* // step size vector*  $q_{t}=\rho ⊙\left(A^{T}\;r_{t}\right)$* // normalized gradient vector*  $\Lambda_{t+1}=\Lambda_{t+1}^{★}$* // initialize the set of sparsity $\Lambda_{t+1}$*  **if**t>0**then**   **while** (stop criterion on $\Lambda_{t+1}$) **do**   ${\tilde{x}}_{t+1}=x_{t}-\mu_{t}\left(\Lambda_{t+1}\right)q_{t}$
* // update $x_{t}$ with step size $\mu_{t}$ given by (46)*   $\Lambda_{t+1}=\underset{k,\Psi }{supp}\;\left({\tilde{x}}_{t+1}\right)$
* // update set of sparsity $\Lambda_{t+1}$*   $x_{t+1}=F\left({\tilde{x}}_{t+1},\Lambda_{t+1}\right)$
* // find sparse vector $x_{t+1}$*
   **end while**  **else**
   ${\tilde{x}}_{t+1}=x_{t}-\mu_{t}\left(I_{N}\right)q_{t}$   ${\left[x_{t+1}\right]}_{k}=F\left({\tilde{x}}_{t+1},\underset{k,\Psi }{supp}\left({\tilde{x}}_{t+1}\right)\right)$
* // find sparse vector $x_{t+1}$*  **end if**
  t=t+1 **end while**

## 4. Comparative Study

To quantify the performance of the CS algorithms previously described a comparative study has been conducted on two different sets of EMG signals, giving rise to case study A and case study B.

A similar study of CS applied to EMG signal was performed in [49]. In that work sparsity is enforced to the signal with a time-domain thresholding technique, and reconstruction SNR is measured with respect to the sparsified signal. In this work, to have an estimation of overall information loss of the signal, after enforcing sparsity with reduced operator ${\left[x\right]}_{k}$ for each basis, we measured SNR with respect to the original signal *x*.

### 4.1. Case Study A

The signals used in this study refer to three different muscles, namely *biceps brachii*, *deltoideus medius*, and *triceps brachii*. They were recorded by the sEMG acquisition set-up described in [16] and following the protocol outlined in [50,51]. The EMG signal was high-pass filtered at 5 Hz and low-pass filtered at 500 Hz before being sampled at 2 kHz. The algorithms were applied to frames of length N=1024, which is a large value enough to limit SNR variations among frames. In the simulations the index *k* and the compression factor CF=M/N, i.e., the inverse of CR, were varied. The performance has been measured based on the following equivalent signal to noise ratio

(48) $$SNR=20\log_{10}\frac{{∥y∥}_{2}}{{∥y-y_{rec}∥}_{2}}\;,$$

where $y_{rec}$ is the reconstruction signal, by averaging the results obtained with different frames.

#### 4.1.1. Basis Selection

**Figure 1:**
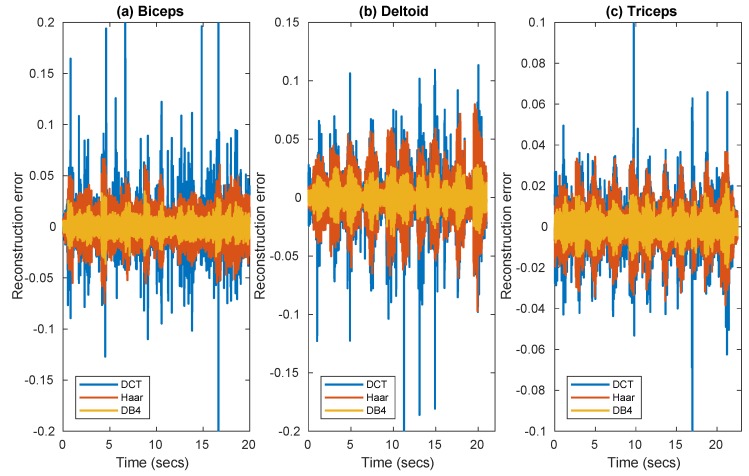
Reconstruction error of EMG signal as achieved with convex optimization, using DCT, Haar, and DB4 bases in frames corresponding to three muscles (**a**) Biceps (**b**) Deltoideus (**c**) Triceps.

Figure 1 compares for the three muscles the reconstruction error in three frames extracted from the data set, as achieved with convex optimization using three different bases, DCT, Haar, and DB4. Since the signal was pre-filtered at 500 Hz, this can lead to an improvement of sparsity in the frequency domain making DCT worth testing.

**Figure 2:**
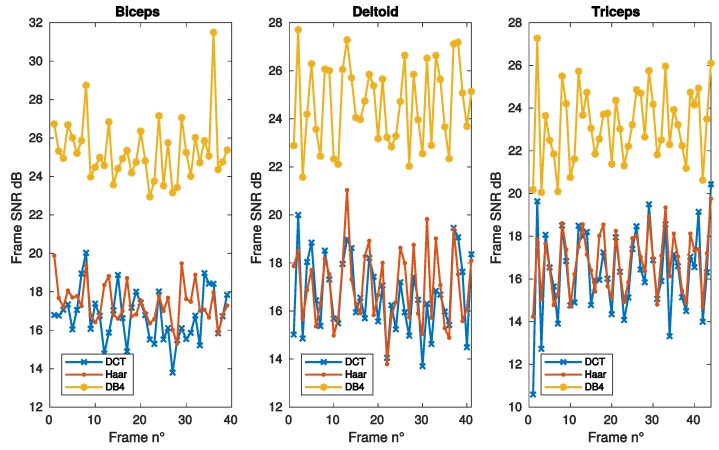
SNR vs. frame number, as achieved with convex optimization, using DCT, Haar, and DB4 bases, for the same muscles of Figure 1.

**Figure 3:**
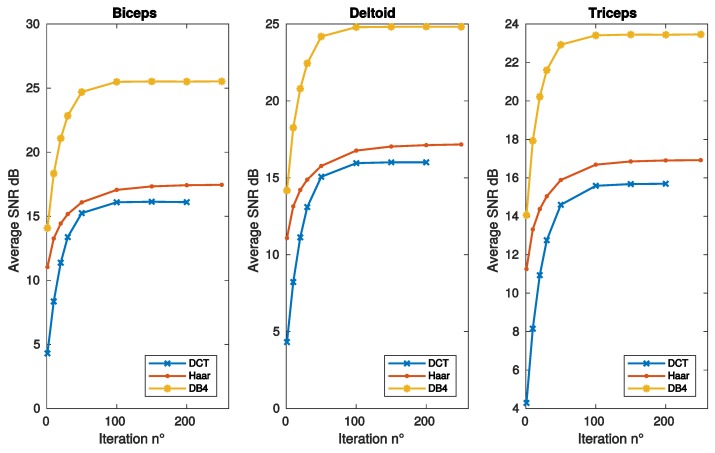
SNR vs. algorithm iterations, as achieved with convex optimization, for the same bases and muscles of Figure 1.

Figure 2 and Figure 3 report the SNR as a function of frame, sparsity and iteration respectively, for the same muscles of Figure 1. DB4 basis clearly shows the best reconstruction performance in all the conditions considered in these figures.

#### 4.1.2. Comparison of Algorithms Performance

As the ultimate goal of this paper is to study and compare the CS methods for the reconstruction of EMG signals, a large experimentation has been carried out with the algorithms previously described.

**Figure 4:**
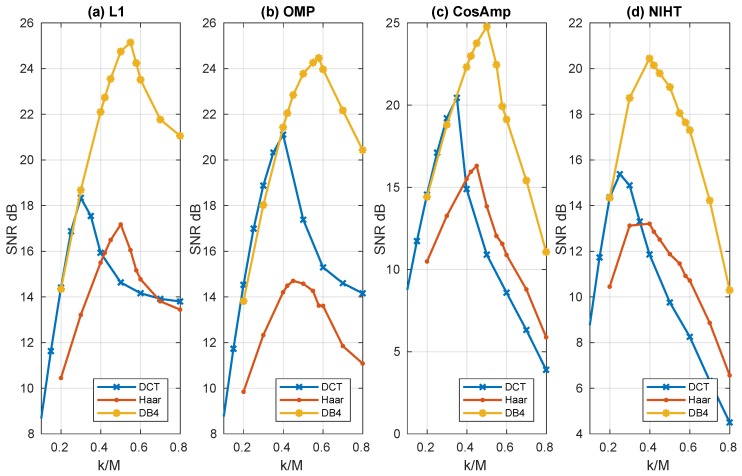
Case Study A—SNR as a function of sparsity $S_{M}=k/M$ with a compression factor CF=0.5, for the four algorithms, (**a**) L1, (**b**) OMP, (**c**) CoSaMP, (**d**) NIHT, using the same bases of Figure 1.

**Figure 5:**
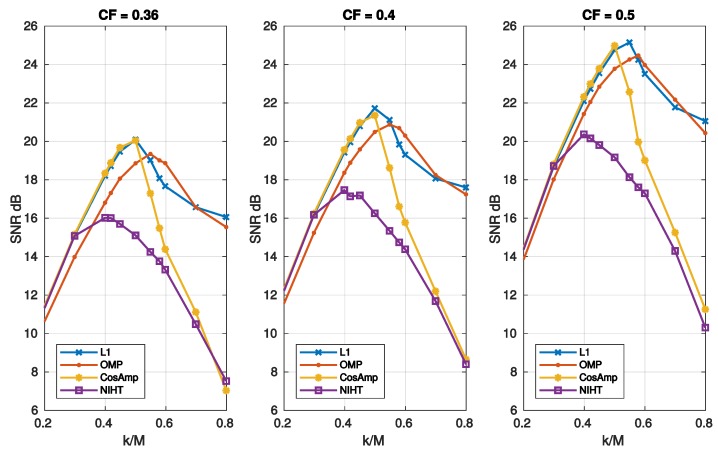
Case Study A—SNR as a function of sparsity $S_{M}=k/M$ and compression factor CF, for the four algorithms, L1, OMP, CoSaMP, NIHT, using the DB4 basis.

**Figure 6:**
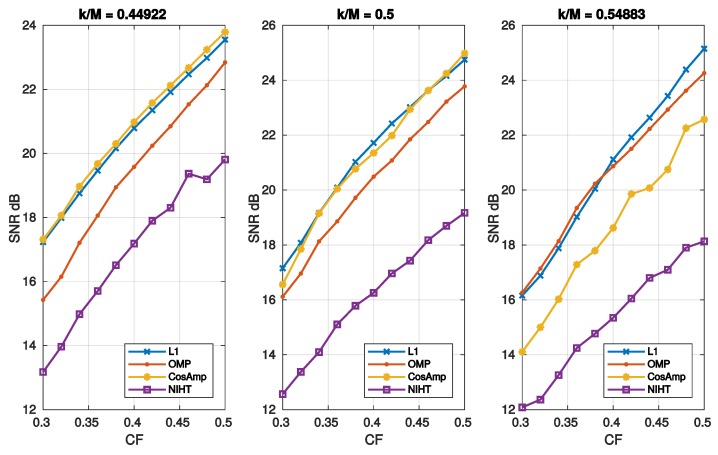
Case Study A—SNR as function of compression factor CF=M/N for the four algorithms and three values of sparsity $S_{M}=k/M$.

**Figure 7:**
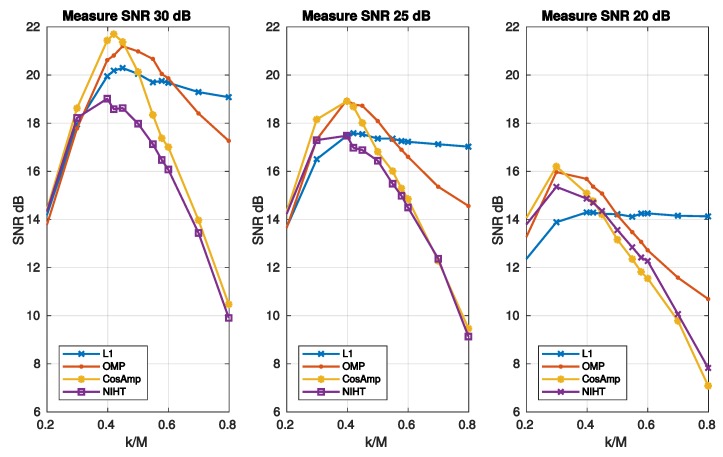
Case Study A—SNR as a function of sparsity $S_{M}=k/M$ for the four algorithms and three values of noise superimposed to the signal.

**Figure 8:**
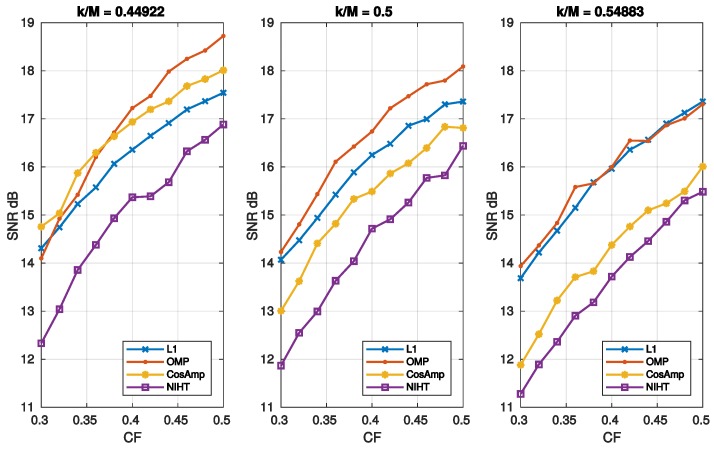
Case Study A—SNR as a function of compression factor CF=M/N for the four algorithms and three values of sparsity $S_{M}=k/M$. A value of SNR = 25 dB for the measurement signal *y* is used.

Figure 4, Figure 5, Figure 6, Figure 7 and Figure 8 report the performance achieved with the four algorithms L1, OMP, CoSaMP, and NIHT under different experimental conditions. In particular the behavior of SNR as a function of sparsity $S_{M}=k/M$ for the four algorithms and the three bases is shown in Figure 4, where a constant value CF=0.5 of compression factor is used. Here the sparsity $S_{M}$ with respect to the dimension *M* has been adopted as for k>M the behavior is not of particular significance. It is evident from these results the superiority of DB4 with respect to other bases as already pointed out in the previously figures. Concerning algorithm performance, all the algorithms show a pronounced peak near the value of k/M=0.4−0.5. This behavior is due to the fact that the SNR is measured with respect to the original signal, and as k/M decreases the fidelity between *x* and ${\left[x\right]}_{k}$ deteriorates. Moreover, while for OMP, CoSaMP, and NIHT, the SNR falls rapidly as k/M increases, the L1 algorithm remains nearly constant beyond the maximum.

Figure 5 reports the SNR as a function of sparsity $S_{M}=k/M$ for different values of CF. In these cases, L1 and OMP have the better performance as they show a similar behavior. Figure 6 depicts the SNR as a function of compression factor CF and different values of $S_{M}$. Also, in this case L1 and OMP show the better performance.

#### 4.1.3. Noise Tolerance

**Figure 9:**
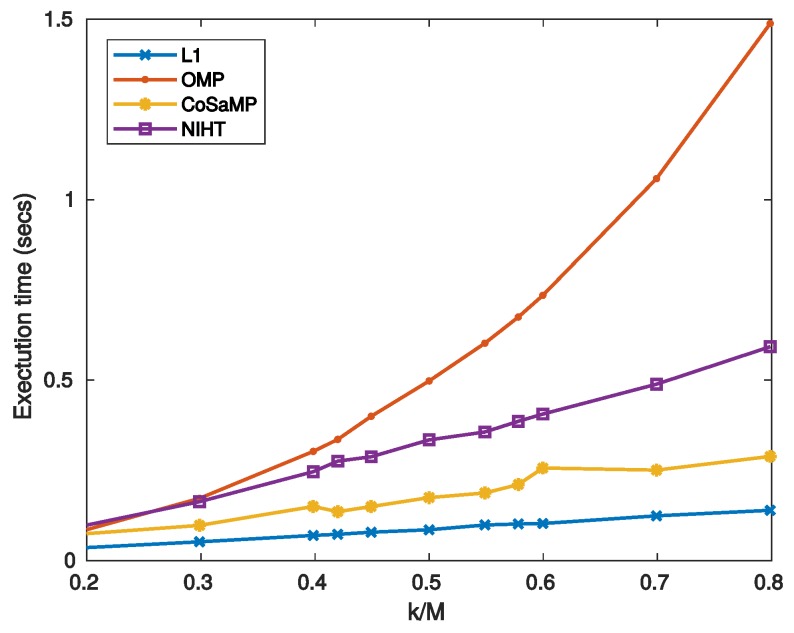
Case Study A—Execution time on MATLAB as a function of sparsity $S_{M}=k/M$.

Real data CS acquisition systems are inherently noisy, thus to simulate a more realistic situation some experiments have been conducted with a noise superimposed to the signal. The effect of a noisy signal *y* on CS reconstruction corresponds to an error $x_{e}$ in the sparse solution *x* given in this case by

(49) $$x=x_{\mathrm{NF}}+x_{e}$$

where $x_{\mathrm{NF}}$ denotes the noise-free solution. The error term $x_{e}$ can be particularized for the four algorithms as follows:

(50) $$\begin{array}{cc} x_{e,L1} & =A^{†}n\\ x_{e,\mathrm{OMP}} & =A_{\Lambda_{k}}^{†}n\\ x_{e,\mathrm{CoS}\mathrm{aMP}} & =A_{T}^{†}n\\ x_{e,\mathrm{NIHT}} & =A_{\Lambda_{0}}^{†}n \end{array}$$

where *n* is the noise superimposed to *y*. It is straightforward to show that the following inequality

(51) $$\frac{∥f-f_{x_{e}}{∥}_{2}}{{∥f∥}_{2}}\geq |\frac{∥f-f_{x_{e}}^{\mathrm{NF}}{∥}_{2}}{{∥f∥}_{2}}-\frac{∥\psi x_{e}{∥}_{2}}{{∥f∥}_{2}}|$$

holds, thus giving the relationship

(52) $$SNR\leq \frac{SNR_{\mathrm{NF}}\;SNR_{\mathrm{noise}}}{SNR_{\mathrm{NF}}-SNR_{\mathrm{noise}}}$$

where $SNR_{\mathrm{NF}}$ and $SNR_{\mathrm{noise}}={∥f∥}_{2}/{∥\psi x_{e}∥}_{2}$ refer to the $x_{\mathrm{NF}}$ and $x_{e}$ components, respectively. For high values of noise SNR degenerates to SNR${}_{\mathrm{noise}}$ thus worsening the noise-free performance. It is worth noticing that for L1 the SNR${}_{\mathrm{noise}}$ is independent of k/M, as it results from Equation (50) and the definition of SNR${}_{\mathrm{noise}}$. This implies that reducing the SNR${}_{\mathrm{noise}}$ does not affect the reconstruction, thus resulting almost constant with k/M. For the other algorithms $x_{e}$ increases with k/M, so that a maximum for the SNR is expected. Figure 7 reports the SNR as a function of sparsity $S_{M}$ for three values of noise superimposed, while Figure 8 is the noisy version of Figure 6, in which a value of SNR = 25 dB for the measurement signal *y* is used. The experimental results confirm the considerations stated above for L1, which shows the worst behavior when the measure SNR decreases. As for OMP, CoSaMP, and NIHT, their performances are almost independent of k/M for low values of it, while suddenly worsen when k/M exceeds a critical value of about 0.5. Finally, Figure 9 reports the computational cost and execution time on MATLAB respectively as functions of sparsity $S_{M}=k/M$. The execution time was computed using MATLAB tic-toc functions. These figures clearly show that L1-minimization outperforms the other algorithms.

### 4.2. Case Study B

The EMG signals used in this case study come from PhysioBank [52], a large and growing archive of well-characterized digital recordings of physiological signals and related data for use by biomedical research community. In particular, the data come from the `Neuroelectric and Myoelectric Databases’ of PhysioBank archives. A class of this database, named `Examples of Electromyograms’ [53], has been used; it contains short EMG recordings from three subjects (one without neuromuscular disease, one with myopathy, one with neuropathy). The signals are sampled at a frequency of 4 kHz and the frame has a length N=1024, the same as case study A. As the signal from this dataset was not low-pass filtered, it contains all typical EMG frequency components, therefore this time we discarded DCT and Haar, using only DB4 basis. We chose to add this case study to analyse performances when signal has the lowest sparsity as possible which is the worst scenario for the reconstruction performance.

**Figure 10:**
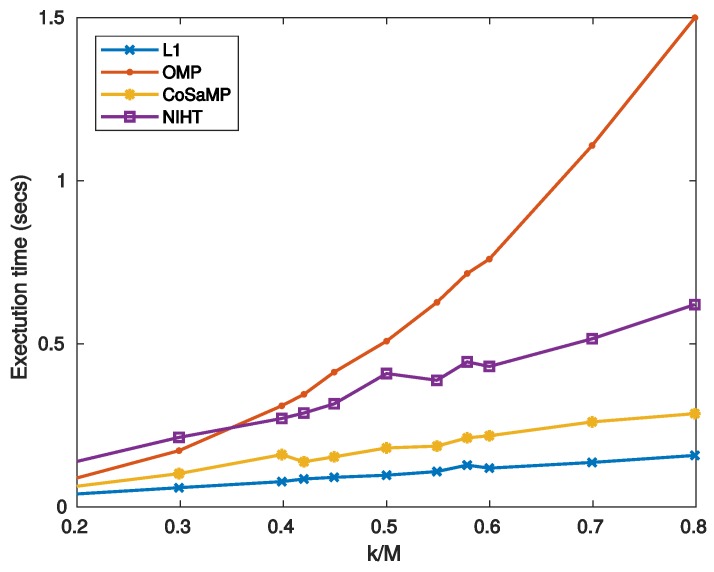
Case Study B—Execution time on MATLAB as a function of sparsity $S_{M}=k/M$.

**Figure 11:**
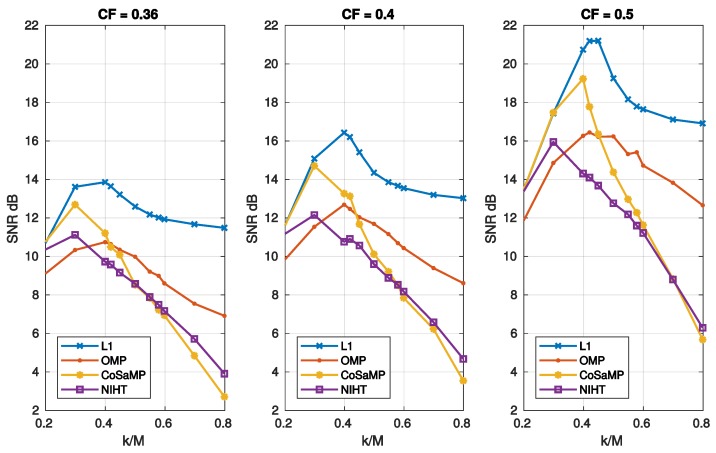
Case Study B—SNR as a function of sparsity $S_{M}=k/M$ and compression factor CF, for the four algorithms, L1, OMP, CoSaMP, NIHT, using the DB4 basis.

Figure 10 reports the execution time as a function of sparsity $S_{M}$ for the four algorithms. Figure 11 compares the SNR as a function of sparsity $S_{M}$ for three values of CF, as obtained with the four algorithms. As shown in these figures, the obtained results have a similar behaviour of those achieved in case study A.

Finally, based on the experimental results previously reported a qualitative assessment of the four reconstruction algorithms can be derived that explores the trade-off in the choice of a CS reconstruction algorithm for EMG sensor application. To this end Table 2 summarizes the performance, in terms of accuracy, noise tolerance, and speed, of the four reconstruction algorithms.

The L1 minimization algorithm has an excellent behavior for accuracy, noise tolerance, and speed, thus outperforming the other algorithms. Among these, CoSaMP shows the best trade-off between accuracy and speed.

## 5. Conclusions

This paper presents a comprehensive comparative study of four of the most common algorithms for CS reconstruction of EMG signals, namely L1-minimization, OMP, CoSaMP, and NIHT. The study has been conducted using a wide range of EMG biosignals coming from two different datasets. Concerning algorithm accuracy, all the algorithms show a pronounced peak of SNR near the value of k/M=0.4−0.5. However, while for OMP, CoSaMP, and NIHT, the SNR falls rapidly, the L1 algorithm remains nearly constant beyond the maximum. As for the effect of noise on CS reconstruction, L1-minimization shows a behavior that is almost independent of k/M. The results on computational cost and execution time on MATLAB show that L1-minimization outperforms the other algorithms. Finally, Table 2 summarizes the performance, in terms of accuracy, noise tolerance, speed, and computational cost of the four algorithms.

## Figures and Tables

**Table 1:** Notation.

Symbol	Description
⊙	element-wise product of two vectors, i.e., $c=a⊙b=\left(a_{1}b_{1},\ldots ,a_{N}b_{N}\right),\;a=\left(a_{1},\ldots ,a_{N}\right),b=\left(b_{1},\ldots ,b_{N}\right)$
⊕	bitwise XOR between two binary arrays
${\left\lfloor x\right\rfloor }_{s_{r}}$	$s_{r}$ samples circular shift of vector *x*
sgn(x)	element-wise sign function of a vector *x*
$B^{†}$	pseudo-inverse of matrix *B*
Λ=supp(x)	support of *x*, the set of indices $\Lambda =\{j:x_{j}\neq 0\}$
|Λ|=k	cardinality of the set Λ (the number *k* of elements in the set)
${\left||x|\right|}_{0}=\left|supp\left(x\right)\right|$	$l_{0}$-norm of *x*
${\left||x|\right|}_{p}=(\sum_{i=1}^{n}|x_{i}{{|}^{p})}^{1/p}$	$l_{p}$-norm of *x* (for some 0<p<∞)
$x_{\Lambda }$	sub-vector of *x* indexed by set Λ
$B_{\Lambda }$	sub-matrix of *B* made by columns indexed by set Λ
$\underset{k,\Psi }{supp}\left(x\right)$	returns a set Λ of *k* indexes corresponding to the largest values $|x_{i}\left||\right|\Psi_{i}{\left|\right|}_{2}$, $\Psi =[\Psi_{1},\ldots ,\Psi_{N}]$
F(x,Λ)	returns a vector with the same elements of *x* in the sub-set Λ and 0 elsewhere
${\left[x\right]}_{k}=F\left(x,\underset{k,\Psi }{supp}\left(x\right)\right)$	reduced operator

**Table 2:** Comparison, in terms of accuracy and speed, of the four algorithms L1, OMP, CoSaMP, NIHT.

Algorithm	Accuracy	Noise Tolerance	Speed	Computational Cost
L1	Excellent	Excellent	Excellent	$O(N^{2}\;N_{\mathrm{iter}})$
OMP	Good	Good	Bad	$O(M\;k^{3})$
CoSaMP	Fair	Fair	Good	$O(M\;k^{2}\;N_{\mathrm{iter}})$
NIHT	Bad	Fair	Fair	O(MNk)
